# Multifunctional ZnO Nanomaterials with Broad-Spectrum Defect-State Absorption for Enhancing the Photocatalytic Degradation of Organic Dyes

**DOI:** 10.3390/ma19081657

**Published:** 2026-04-21

**Authors:** Ai Zhou, Hongyun Li, Jie Fang

**Affiliations:** 1 Food and Pharmacy College, Zhejiang Ocean University, Zhoushan 316022, China; zhouai9982@126.com; 2 Suzhou Ultra-Nano Technology Co., Ltd., Suzhou 215000, China; leejike52@gmail.com

**Keywords:** zinc oxide, morphology control, broad-spectrum absorption, oxygen vacancy, photocatalytic degradation

## Abstract

Zinc oxide (ZnO) nanomaterials have attracted widespread attention from researchers due to their morphology-dependent properties, eco-friendly characteristics, and potential as a sustainable photocatalyst with a broad range of applications. Therefore, in this study, three different ZnO nanostructures—nanosheets (NSs), nanoflowers (NFs), and nanorods (NBs)—were synthesized via a controlled precipitation method. Among these, NFs exhibited the highest photocatalytic efficiency. The obtained samples exhibited broad optical absorption edges extending into the visible region (corresponding to apparent energies of 1.81–2.09 eV), which is attributed to the sub-bandgap states induced by oxygen vacancies rather than intrinsic bandgap narrowing—far lower than the bandgap of bulk ZnO (3.37 eV). Their photocatalytic performance was evaluated by the degradation of Methyl Blue (MB), Methyl Orange (MO), and Rhodamine B (RhB) under UV or sunlight. Notably, the NFs achieved rapid degradation of MB and RhB within 90 min under UV irradiation without the addition of any H_2_O_2_, demonstrating their effectiveness and cost-effectiveness for practical applications. Although H_2_O_2_ inhibited the degradation of MB and RhB, it promoted the decomposition of MO. Furthermore, the ZnO NFs exhibited excellent recyclability in five consecutive degradation cycles. The self-synthesized ZnO nanomaterials in this study, with their broad-spectrum absorption, high stability, and eco-friendly properties, demonstrate their potential as an efficient and low-cost photocatalyst for large-scale wastewater treatment.

## 1. Introduction

In recent years, the synthesis of semiconductor nanomaterials has attracted widespread attention. Their excellent size-dependent, optical, and electronic properties hold great promise for numerous applications [1]. As one of the most favored nanomaterials [2], ZnO is widely used in electronic sensors [3], gas sensors [4,5], coatings [6], photodegradation [7], cosmetics [8], and biomedicine [9]. As a typical n-type II-VI oxide semiconductor, it possesses a large bandgap (3.37 eV) and a high exciton binding energy (60 meV), and forms electron–hole pairs under ultraviolet irradiation [10]. Currently, TiO_2_ is the most commonly used photocatalyst in water hydrolysis or dye degradation. Although TiO_2_ is the most widely used photocatalyst, it suffers from rapid electron–hole recombination and a limited visible light response. Recent efforts have focused on engineering TiO_2_-based heterojunctions to overcome these drawbacks [11]. ZnO is considered the best alternative to TiO_2_ [12], the primary reasons being that ZnO’s bandgap energy is close to that of TiO_2_ [13], ZnO exhibits activity similar to or stronger than TiO_2_ [14], and ZnO has lower synthesis costs and higher efficiency. Furthermore, ZnO-based nanomaterials with high specific surface area and high activity demonstrate excellent antibacterial potential in suppressing various pathogens. Their novel properties and low material consumption have attracted attention from various disciplines and industries worldwide. Today, ZnO nanomaterials are widely used in the food packaging industry for their ability to inhibit and control microbial growth, retain moisture, prevent penetration of liquids or gases, and control shelf life [15]. In addition, nanotechnology is very rapidly entering the textile industry [16], the pharmaceutical industry, etc.

Of course, the different morphologies of ZnO can have an impact on its properties. For example, it has been found that by varying the mixing of precursors, different morphologies of particles (spherical, hexagonal and nanosheets) were synthesized and the photocatalytic activity of the prepared ZnO NPs was demonstrated to be superior to other morphologies by probing the degradation of Rhodamine B (RhB) in water under visible light irradiation [17]. Wan et al. prepared ZnO nanowires for the first time using chemical vapor deposition and verified that ZnO nanowire gas sensors display high sensitivity and a fast response to ethanol gas at 300 °C [18]. Li et al. prepared multilayer ZnO nanosheets with a three-dimensional porous structure by calcination and found that ZnO nanosheets are very superior for the preparation of optoelectronic devices and gas sensors [19]. Researchers have reported several methods for the synthesis of ZnO nanoparticles, including precipitation [20], chemical techniques [21], sol–gel methods [22], hydrothermal methods [23], solvothermal methods [24] and microwave techniques [25]. For example, Yang et al. successfully prepared a new type of porous ZnO nanosheets using hydrothermal and calcination methods in their study. The gas-sensitive performance test results show that the ZnO sensor has good sensitivity and stability to lower concentrations of CH_4_ at lower operating temperatures, effectively improving the gas-sensitive performance of the reported CH_4_ sensor [26]. And, Koutavarapu et al. used a simple and environmentally friendly hydrothermal method to synthesize Bi_2_WO_6_/ZnO photocatalysts, which can significantly improve the photodegradation of Rhodamine B (RhB) under solar light irradiation [27]. However, most of these methods involve complex, time-consuming steps, and/or the use of organic solvents or surfactants [28]. With the concept of green chemistry gradually becoming rooted in the hearts of the people, researchers worldwide have done a lot of research on the green synthesis of ZnO nanoparticles [29]. This includes the use of many plant materials and microorganisms that are harmless to the environment to avoid the use of toxic chemicals [30]. Many authors have used different techniques to synthesize various ZnO nanostructures. For example, Vidya C et al. utilized the biological components of Tribulus terrestris leaf extract for the green synthesis of ZnO nanocrystals using zinc nitrate. ZnO with an average particle size of 30~35 nm was synthesized using a rapid, simple and environmentally friendly method [31]. Sagar Raut et al. synthesized hexagonal ZnO nanoparticles with diameters ranging from 11~25 nm using the leaves of Garcinia Cambogia plant as a reducing agent [32]. In addition, Sagar Raut et al. used fresh alfalfa leaves as a reducing and stabilizing agent using a biological method to synthesize NPs with an average particle size between 15~25 nm [33].

Here, we report an environmentally friendly and scalable method for synthesizing ZnO nanomaterials via “process intensification” (PI). This method is a continuous, room-temperature process that requires only a few seconds and does not involve the use of any surfactants. We have successfully prepared uniform ZnO nanomaterials with various morphologies and evaluated the differences in their photodegradation performance. Unlike conventional ZnO with UV-only activity, our ZnO nanostructures exhibit defect-engineered visible light absorption, enabling solar-driven degradation without external additives.

## 2. Experimental Section

### 2.1. Materials

Zinc chloride (analytical grade, ≥98%, China National Pharmaceutical Group Chemical Reagents Co., Ltd., Beijing, China); zinc sulfate heptahydrate (analytical grade, ≥98%, China National Pharmaceutical Group Chemical Reagents Co., Ltd.); Methylene Blue (analytical grade, ≥98%, China National Pharmaceutical Group Chemical Reagents Co., Ltd.); Methyl Orange (analytical grade, ≥98%, China National Pharmaceutical Group Chemical Reagents Co., Ltd.); Rhodamine B (analytical grade, ≥98%, China National Pharmaceutical Group Chemical Reagents Co., Ltd.); anhydrous ethanol (analytical grade, ≥98%, China National Pharmaceutical Group Chemical Reagents Co., Ltd.); sodium hydroxide (analytical grade, ≥98%, China National Pharmaceutical Group Chemical Reagents Co., Ltd.); deionized water.

### 2.2. Synthesis

By varying the flow rate, rotation speed, zinc source, and solution concentration during the synthesis process, we successfully prepared three zinc oxide nanomaterials with different morphologies. The chemical equation for this reaction is Zn^2+^ + 2OH^−^ → Zn(OH)_2_ → ZnO + H_2_O. The following is a detailed preparation process.

NSs were synthesized using the direct precipitation method. Solid zinc chloride was dissolved in water to prepare a 0.05 mol/L solution, and solid sodium hydroxide was dissolved in water to prepare a 0.15 mol/L solution. The total reaction volume was 2 L, with a zinc chloride feed rate of 13.6 g and a sodium hydroxide feed rate of 12.0 g. The peristaltic pump flow rate was adjusted to 40 mL/min, and the reactor turntable speed was adjusted to 1000 rpm. The precipitate obtained after the reaction was dried overnight at 120 °C to obtain the final product.

NFs were synthesized using the direct precipitation method. Zinc sulfate heptahydrate solid was dissolved in water to prepare a 0.15 mol/L aqueous solution, and sodium hydroxide solid was dissolved in water to prepare a 0.45 mol/L aqueous solution. The total reaction volume was 2 L, with a zinc sulfate heptahydrate feed rate of 86.3 g and a sodium hydroxide feed rate of 36.0 g. The peristaltic pump flow rate was adjusted to 100 mL/min, and the reactor disk speed was adjusted to 1500 rpm. The precipitate obtained after the reaction was dried overnight at 120 °C to obtain the final product.

NBs were synthesized using the direct precipitation method. Solid zinc chloride was dissolved in anhydrous ethanol to prepare a 0.01 mol/L solution, and solid sodium hydroxide was dissolved in anhydrous ethanol to prepare a 0.04 mol/L solution. The total reaction volume was 2 L, with a zinc chloride feed rate of 2.7 g, sodium hydroxide was added at a rate of 3.2 g, the peristaltic pump flow rate was adjusted to 40 mL/min, and the reactor turntable speed was adjusted to 1000 rpm. The precipitate obtained after the reaction was dried overnight at 120 °C to obtain the final product.

The three morphologies described above were synthesized independently in three batches; the SEM and XRD results were consistent.

**Figure 1:**
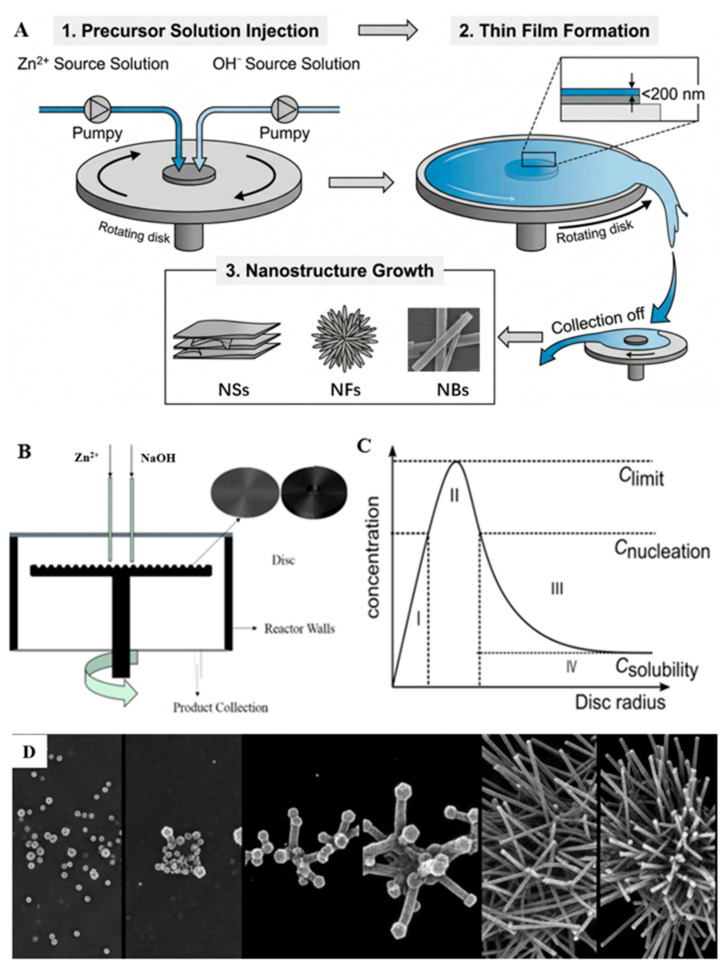
(**A**) Schematic illustration of the continuous flow synthesis procedure, (**B**) schematic view of a spinning disk reactor, (**C**) reaction mechanism, (**D**) growth process of zinc nanostructures.

Figure 1 shows the continuous flow reactor with thin film (CFFR) and the ZnO synthesis mechanism [34], which continuously injects the reaction liquid into a high-speed rotating surface. Under centrifugal force, the reaction fluid is rapidly drawn into a thin film smaller than 200 nm. The shear stress generated by the rotation of the surface texture maximizes the mass and heat transfer efficiency of the thin film reaction fluid.

### 2.3. Characterization

Ultraviolet-Visible Spectrophotometer (Model: UV-2600, Shimadzu Corporation, Kyoto, Japan); Ultrasonic Cleaner (Model: KQ-250DE, Kunshan Ultrasonic Instrument Co., Ltd., Kunshan, China); magnetic stirrer (Model: 85-2, Shanghai Jingke Instrument Co., Ltd., Shanghai, China); Constant Temperature Water Bath (Model: HH-4, Jintan Jierui Electrical Appliances Co., Ltd., Jintan, China); forced-air drying oven (Model: DHG-9070A, Shanghai Jinghong Experimental Equipment Co., Ltd., Shanghai, China); scanning electron microscope (Model: S-4800, Hitachi Co., Ltd., Tokyo, Japan); X-ray diffractometer (Model: D8 Advance, Bruker Corporation, Karlsruhe, Germany).

Observe the morphology and size of zinc oxide nanomaterials using a scanning electron microscope (SEM). The accelerating voltage is 10–15 kV, and the working distance is 8–12 mm. Analyze the crystal structure and phase composition of zinc oxide nanomaterials using an X-ray diffractometer (XRD); and detect the effective wavelength of zinc oxide nanomaterials using a UV-Vis Spectrophotometer (Shimadzu, Kyoto, Japan).

### 2.4. Photodegradation Experiment

#### 2.4.1. Experimental Facility

A homemade photocatalytic reaction apparatus was used, consisting of three quartz glass reactors, a 250-watt ultraviolet high-pressure mercury lamp as the light source, a set of 300-watt long-arc xenon lamp devices, three cooling devices, and a quadruple magnetic stirrer. During the reaction process, the reaction solution was continuously stirred by the magnetic stirrer to ensure sufficient contact between the reaction solution and the zinc oxide nanomaterials. In this experiment, the pH was maintained within the range of 6.8–7.2. The light intensity for the UV experiment corresponded to the power of the purchased high-pressure mercury lamp, which was 250 watts. For the sunlight experiment, a solar simulator was used, employing a purchased long-arc xenon lamp with a power of 300 watts.

#### 2.4.2. Experimental Methods

##### Effect of H_2_O_2_ on Photodegradation Efficiency

MB, MO, and RhB were prepared as organic dye solutions with a concentration of 2 mg/mL. 95 mL of pure water was added to each of the four reaction glassware containers, followed by 30 mg of NSs, NFs and NBs powder, respectively. The solutions were sonicated to ensure uniform dispersion. Then, 4 mL of H_2_O_2_ (3%) and 1 mL of MB/MO/RhB were added, resulting in a total reaction volume of 100 mL. Magnetic stir bars were added to the reaction vessels, which were placed on a four-station magnetic stirrer set at 300 rpm. Prior to the formal reaction, stirring was conducted in the dark for 5 min to achieve adsorption–desorption equilibrium. After the dark treatment, the reaction is carried out under the irradiation of a 250-watt ultraviolet high-pressure mercury lamp. Samples are taken every 15 min, with the height between the reactor and the light source controlled at 23 cm. To prevent changes in the total reaction volume due to a temperature rise during the reaction, a cooling device should be prepared in advance. When the colored solution in the reaction flask turns colorless, the reaction is considered complete, and the reaction is stopped. During the reaction, samples are centrifuged using a high-speed centrifuge at 10,000 rpm for 10 min to separate the nanomaterials from the solution. The supernatant is then measured for absorbance using an enzyme-linked immunosorbent assay (ELISA) reader. The detection wavelengths are 664 nm for MB, 465 nm for MO, and 554 nm for RhB. The degradation rate is calculated based on the measured absorbance. The degradation rate formula is: (1 − At/A0) × 100%, where A0 is the absorbance of the substance to be degraded at the initial time (t = 0), and At is the absorbance of the substance to be degraded at time t. The above is the experimental procedure for experiments containing H_2_O_2_. To compare the effect of hydrogen peroxide on the photodegradation of zinc oxide nanomaterials, additional experiments without H_2_O_2_ should be conducted, following the same experimental procedure.

##### The Effect of Cycle Count on Photodegradation Efficiency

Based on the photodegradation efficiency of ZnO nanomaterials with different morphologies, we conducted a data analysis. Here, we selected NFs as the primary material for the photodegradation cycling experiments to test the effect of multiple cycles on photodegradation efficiency.

Solutions of MB and RhB were prepared at a concentration of 2 mg/mL. One milliliter of each solution was added to the ultrasonically dispersed NF solution, resulting in a total reaction volume of 100 mL. A magnetic stir bar was added, and the mixture was placed on a magnetic stirrer set to 300 rpm. At the start of the reaction, dark adsorption was first performed under light-shielded conditions to allow the organic dyes to fully adsorb onto the surface of the nanomaterials, followed by irradiation under a 250-watt high-pressure mercury UV lamp. Samples were collected every 20 min. The reaction was terminated when the colored solution in the reaction flask turned colorless. The samples collected during the reaction were centrifuged, and the supernatant was analyzed using a microplate reader. The detection wavelength for MB was 664 nm, and for RhB, it was 554 nm. The degradation rate was calculated based on the measured absorbance values.

## 3. Results and Discussion

### 3.1. Material Characterization

**Figure 2:**
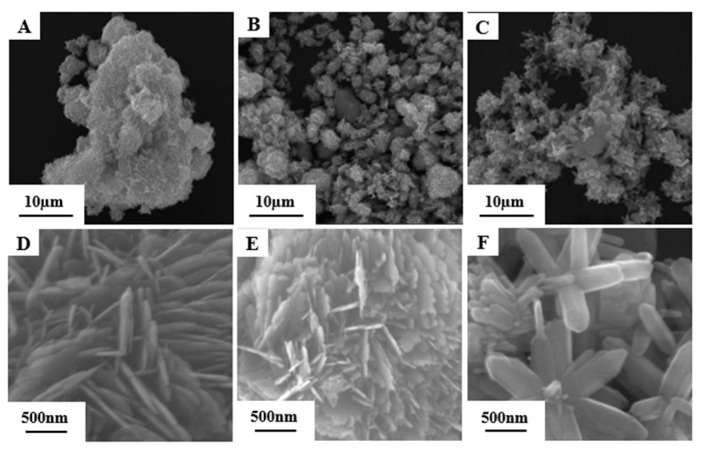
SEM images of ZnO nanostructures at different magnifications. Low-magnification overview (scale bar: 10 µm): (**A**) nanosheets, (**B**) nanoflowers, (**C**) nanobars. High-magnification details (scale bar: 500 nm): (**D**) nanosheets, (**E**) nanoflowers, (**F**) nanobars.

Multiple regions of the synthesized ZnO samples were imaged using SEM, and representative images are presented in Figure 2. The morphology of the synthesized ZnO samples was examined by SEM. As shown in Figure 2A–C, three distinct nanostructures were obtained, NSs, NFs and NBs. As shown in Figure 2A,C,D,F, the samples prepared using ZnCl_2_ exhibit hierarchical sheet-like and rod-like microsphere morphologies. These microspheres have a uniform morphology and are assembled from densely arranged nanosheets and nanorods radiating outward from the center. Statistical analysis based on multiple images indicates that the average diameters of these microspheres are 4.87 ± 0.31 µm and 2.87 ± 0.51 µm. The nanosheets and nanorods constituting these microspheres have smooth surfaces, with an average thickness of 9.7 ± 2.5 nm and an average length of 553.3 ± 41.6 nm.

In contrast, samples synthesized using ZnSO_4_ (Figure 2B,E) exhibit flower-like microspheres, which are irregular aggregates with an average size of 2.13 ± 0.31 µm.

The formation of these different morphologies depends largely on the nature of the anions in the zinc precursor. Cl^−^ ions are believed to adsorb specifically onto the nonpolar faces of ZnO nuclei, effectively suppressing the lateral growth rate while allowing rapid elongation along the c-axis. This anisotropic growth mechanism favors the formation of nanorods. In contrast, the presence of SO_4_^2−^ ions introduces different electrostatic interactions and steric hindrance effects, leading to less ordered aggregation.

**Figure 3:**
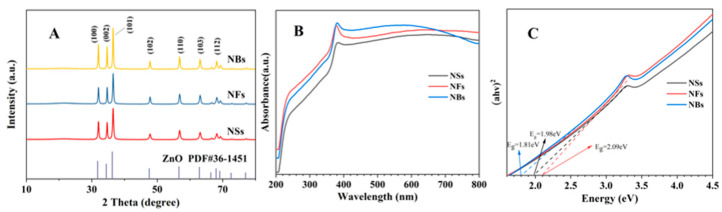
(**A**) XRD patterns of the synthesized ZnO nanostructures, (**B**) UV-Vis absorption spectra, (**C**) Tauc plots.

The XRD pattern (Figure 3A) shows peaks at 2θ = 31.8° (100), 34.4° (002), 36.3° (101), 47.5° (102), 56.6° (110), 62.9° (103), and 68.0° (112), consistent with the standard hexagonal zinc oxide (Wurtzite) structure (JCPDS No. 36-1451). No impurity phases such as cubic ZnO or Zn(OH)_2_ were detected, confirming the phase purity of all morphologies. A comparison of the three different XRD patterns revealed no significant differences among them. Based on XRD phase purity, impurity-induced absorption is unlikely; the sub-bandgap absorption is attributed to oxygen vacancies.

Based on our analysis of the absorption spectra, the data indicate a red shift in the absorption edges. The positions of the absorption edges, calculated using the formula λ = 1240/Eg, are approximately 685 nm (Eg = 1.81 eV), 626 nm (Eg = 1.98 eV), and 594 nm (Eg = 2.09 eV). The steepness of the absorption edge reflects the uniformity of the sample size. The strong absorption peaks in the 200–400 nm wavelength range originate from intrinsic band-edge transitions in ZnO (O → Zn). Samples with smaller Eg values exhibit enhanced absorption at longer wavelengths (>600 nm), which is most likely due to sub-bandgap absorption caused by defect states or surface states.

ZnO is a wide-bandgap semiconductor (3.37 eV for bulk), and its optical bandgap (Eg) and UV absorption are strongly influenced by morphology and size. In this study, as can be seen from the Tauc plots in Figure 3C, the bandgaps of the three ZnO morphologies were derived from UV-Vis absorption spectra, yielding values of 1.81 eV, 1.98 eV, and 2.09 eV for NBs, NSs and NFs respectively. All three nanostructures showed Eg values much lower than that of bulk ZnO, which may be attributed to the quantum size effect and the presence of defect states such as oxygen vacancies (OVs) [35]. Therefore, the Eg value obtained from the Tauc plot may not represent the true bandgap, but rather the apparent optical bandgap (which includes contributions from defect states); the actual bandgap remains close to 3.3 eV. Future research may further elucidate defect states through photocurrent spectroscopy [36]. An Eg of ~2.0 eV corresponds to an absorption edge near 620 nm, within the orange–red visible region, implying that these ZnO nanomaterials can absorb visible light, unlike conventional ZnO which is limited to UV absorption. This visible light response offers advantages for photocatalysis, photovoltaics, and bioimaging.

### 3.2. Photocatalytic Performance Analysis

**Figure 4:**
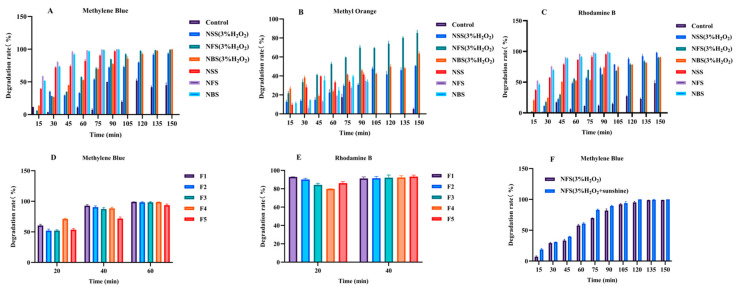
(**A**–**C**) Degradation of MB, MO and RhB by different forms of ZnO at different time points, (**D**,**E**) five-cycle degradation of MB and RhB by ZnO NFs, (**F**) degradation of MB by NFs under ultraviolet or sunlight.

Figure 4A–E presents the photodegradation results of MB, MO, and RhB under UV irradiation. The photocatalytic activity of the three ZnO morphologies followed the order NFs > NBs > NSs. Notably, the degradation rates were generally higher in the absence of H_2_O_2_. This is because H_2_O_2_ can consume photogenerated electrons, disrupt reactive oxygen species (ROS) generation pathways, and quench •OH radicals at high concentrations. Without H_2_O_2_, ZnO’s intrinsic ROS generation mechanism operates more synergistically and efficiently. In contrast, some reported systems (e.g., Fe_2_O_3_/UV and TiO_2_/UV/O_3_/H_2_O_2_) show enhanced degradation with H_2_O_2_ due to additional •OH generation and suppressed carrier recombination [37,38].

For MB degradation (Figure 4A), NFs achieved about 60% removal within 15 min without H_2_O_2_, while with H_2_O_2_ the efficiency dropped to ~5% at the same time point. Complete degradation with H_2_O_2_ required 60 min longer than without. Thus, H_2_O_2_ inhibited MB degradation under these conditions. Additionally, MB exhibited noticeable self-degradation under UV light, likely due to photoreactive functional groups in its structure that undergo photochemical reactions such as redox processes and intramolecular rearrangements. The emission spectrum of the mercury lamp, rich in UV and blue light, overlaps with the absorption profile of MB, further promoting its photolysis.

For MO degradation (Figure 4B), NFs still showed the highest efficiency, but overall degradation was slower compared to MB. After 150 min, MO degradation did not reach 100%, whereas MB was completely degraded within 90 min. Interestingly, H_2_O_2_ enhanced MO degradation, contrary to its effect on MB. This reversal may be attributed to the presence of azo bonds and sulfonic acid groups in MO, which are highly susceptible to oxidation by •OH radicals generated from H_2_O_2_. In contrast, MB’s molecular structure is more stable and less sensitive to •OH attack. MO exhibited minimal self-degradation, owing to the relative stability of its azo bonds under the given irradiation conditions, the protective role of sulfonic acid groups.

For RhB degradation (Figure 4C), the trend resembled that of MB, with >50% removal within 15 min and complete degradation around 90 min. Self-degradation was also observed, likely due to the photoactive anthracene ring and substituents in RhB that facilitate excitation, redox reactions, free radical formation, and intramolecular rearrangement under UV light.

Based on the above photodegradation results, we observed an interesting phenomenon: why H_2_O_2_ inhibits the degradation of MB and RhB but promotes the degradation of MO. This may be because, for MB and RhB, H_2_O_2_ may act as a recombination center at high concentrations, consuming conduction band electrons (e^−^) and preventing the formation of ·O^2−^, thereby inhibiting degradation.

**Figure 5:**
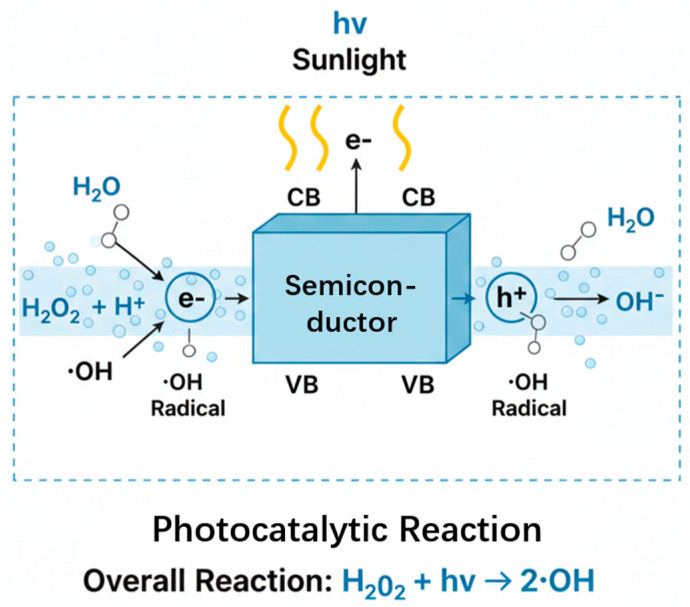
Schematic diagram of the photocatalytic reaction mechanism.

For MO, the azo bonds (-N=N-) are more susceptible to cleavage by hydroxyl radicals (•OH). H_2_O_2_ provides an additional source of •OH (H_2_O_2_ + e^−^ → •OH + OH^−^), thereby promoting degradation. For a detailed schematic diagram, please refer to Figure 5.

In cyclic degradation experiments using NFs (Figure 4D,E), their excellent reusability was demonstrated. Over five consecutive cycles, the degradation rates of MB and RhB remained above 50% and 80%, respectively, within the first 20 min, and near-complete degradation was achieved within 60 min in each cycle. These results confirm the high structural stability and practical application potential of NFs as reusable photocatalysts.

In Figure 4F, we selected MB as the degradation target and NFs as the degradation medium. The figure shows that, with all other conditions held constant, changing the light source revealed that NFs exhibited superior degradation performance under sunlight compared to UV light. This result further confirms that this material possesses strong absorption characteristics in the visible spectrum, providing important data support for the use of sunlight in the treatment of organic wastewater.

Based on the degradation rate results above, we also developed a Langmuir–Hinshelwood (L-H) kinetic model. As shown in Table 1, the kinetic parameters were calculated based on the pseudo-first-order model.

Activity trend: The reaction rate constant (K) follows the order of NBs > NFs > NSs, indicating that the nanorod morphology possesses superior catalytic activity.

Effect of H_2_O_2_: Surprisingly, the addition of H_2_O_2_ resulted in a significant decrease in the K values for all samples. For instance, the K value of NBs for RhB degradation dropped from 0.0438 min^−1^ to 0.0143 min^−1^ upon adding H_2_O_2_. This suggests that H_2_O_2_ acts as a scavenger of active species or competitively adsorbs on the active sites, thereby inhibiting the catalytic process rather than promoting it.

Substrate dependency: The catalysts showed the highest efficiency towards RhB degradation, followed by MB, while MO was the most recalcitrant, as evidenced by the lowest K values (e.g., 0.0047 min^−1^ for NBs). This indicates a strong dependence on the chemical structure and charge of the dye molecules.

## 4. Conclusions

Based on the above studies, it is evident that morphological engineering is an effective strategy for modulating the photocatalytic performance of ZnO nanomaterials. In this study, we synthesized three ZnO nanomaterials with distinct morphologies—NSs, NFs, and NBs—via a precipitation method, with bandgaps tunable between 1.81 eV and 2.09 eV. NFs exhibited excellent activity toward all three dyes (MB, MO, and RhB). Compared to pristine ZnO (3.37 eV), the materials synthesized in this study possess stronger visible light absorption capacity; however, they did not demonstrate enhanced degradation efficiency in the presence of hydrogen peroxide. This characteristic indicates that in practical wastewater treatment applications, efficient degradation can be achieved with sunlight alone, without the need for UV irradiation or additional additives. The NFs maintained high photocatalytic stability over five cycles, with a degradation efficiency of over 80% for RhB and over 50% for MB within 20 min. In summary, ZnO nanoflowers have emerged as an efficient, recyclable, and cost-effective photocatalyst for the removal of organic dyes. This morphology-based bandgap engineering approach offers a viable strategy for optimizing ZnO to achieve broad-spectrum, solar-driven water purification. Future research should focus on immobilizing the nanoflowers on suitable supports to enable operation in continuous flow reactors and investigate their photocatalytic mechanisms under natural sunlight.

## Figures and Tables

**Table 1:** Pseudo-first-order kinetic parameters (K and R^2^) for the degradation of different dyes over samples with various morphologies.

Sample	Dye	H_2_O_2_	K (min^−1^)	R^2^
NSs	MB	Yes	0.0184	0.9335
		No	0.0325	0.9614
	MO	Yes	0.0045	0.9234
		No	0.0049	0.6698
	RhB	Yes	0.0292	0.8878
		No	0.0349	0.9922
NFs	MB	Yes	0.0362	0.9463
		No	0.0606	0.8697
	MO	Yes	0.0125	0.9685
		No	0.0061	0.9677
	RhB	Yes	0.0155	0.9353
		No	0.0511	0.9829
NBs	MB	Yes	0.0364	0.8489
		No	0.0718	0.9891
	MO	Yes	0.0044	0.6593
		No	0.0047	0.6015
	RhB	Yes	0.0143	0.9229
		No	0.0438	0.9832

K represents the pseudo-order reaction rate constant. R^2^ represents the correlation coefficient.

## Data Availability

The original contributions presented in this study are included in the article. Further inquiries can be directed to the corresponding author.
